# One-step parametric network meta-analysis models using the exact likelihood that allow for time-varying treatment effects

**DOI:** 10.1017/rsm.2025.21

**Published:** 2025-05-15

**Authors:** Harlan Campbell, Dylan Maciel, Keith Chan, Jeroen P. Jansen, Sven Klijn, Kevin Towle, Bill Malcolm, Shannon Cope

**Affiliations:** 1 Health Economics and Outcomes Research, Precision AQ, Vancouver, BC, Canada; 2 Bristol Myers Squibb, Princeton, NJ, USA; 3 Bristol Myers Squibb, Uxbridge, UK

**Keywords:** comparative effectiveness research, evidence synthesis, network meta-analysis, parametric models, time-to-event outcomes

## Abstract

The importance of network meta-analysis (NMA) methods for time-to-event (TTE) that do not rely on the proportional hazard (PH) assumption is increasingly recognized in oncology, where clinical trials evaluating new interventions versus standard comparators often violate this assumption. However, existing NMA methods that allow for time-varying treatment effects do not directly leverage individual events and censor times that can be reconstructed from Kaplan–Meier curves, which may be more accurate than discrete hazards. They are also challenging to implement given reparameterizations that rely on discrete hazards. Additionally, two-step methods require assumptions regarding within-study normality and variance. We propose a one-step fully Bayesian parametric individual patient data (IPD)-NMA model that fits TTE data with the exact likelihood and allows for time-varying treatment effects. We define fixed or random effects with the following distributions: Weibull, Gompertz, log-normal, log-logistic, gamma, or generalized gamma distributions. We apply the one-step model to a network of randomized controlled trials (RCTs) evaluating multiple interventions for advanced melanoma and compare results with those obtained with the two-step approach. Additionally, a simulation study was performed to compare the proposed one-step method to the two-step method. The one-step method allows for straightforward model selection among the “standard” distributions, now including gamma and generalized gamma, with treatment effects on either the scale alone or with multivariate treatment effects. Generalized gamma offers flexibility to model U-shaped hazards within a network of RCTs, with accessible interpretation of parameters that simplifies to exponential, Weibull, log-normal, or gamma in special cases.

## Highlights

### What is already known

Network meta-analysis (NMA) methods that do not rely on the proportional hazard assumption (for time-to-event outcomes) are often required in oncology. Individual event and censor times from clinical trials can typically be reconstructed from published Kaplan–Meier curves, but existing NMA methods used to synthesize these trials do not directly leverage this individual-level data.

### What is new

We propose using a one-step fully Bayesian parametric NMA model that leverages individual event and censor times with the exact likelihood and allows for time-varying treatment effects. By using the exact likelihood specification, this model offers a flexible fit while avoiding certain assumptions/approximations required with existing approaches. The Bayesian framework ensures that model selection and interpretation is straightforward and efficient computation for fitting the model can be done using Stan software.

### Potential impact for RSM readers outside the authors’ field

Flexible NMA methods are particularly important when there are differences in the survival distributions for the different treatments being compared, and relative treatment effects need to be extrapolated beyond the available trial data for a cost-effectiveness analysis. In this context, the one-step parametric NMA model offers a valuable tool that leverages individual-level data.

## 1 Introduction

Network meta-analysis (NMA) is a widely used statistical technique that expands upon standard pairwise meta-analysis, enabling simultaneous direct and indirect comparisons of multiple interventions within a unified statistical model.^1^^–^ ^3^ Given that randomized controlled trials (RCTs) in oncology rarely compare the new intervention to all relevant comparators, NMAs are often needed to synthesize time-to-event outcomes (TTE), such as progression-free survival (PFS) and overall survival (OS).^4^

The synthesis of TTE outcomes is typically based on hazard ratios (HRs)^5^ derived from the Cox proportional hazards (PH) model.^6^^,^ ^7^ However, for many reasons, the PH assumption may be implausible,^8^^–^ ^10^ for example, due to treatment effects that vary over time, treatment crossover, delayed treatment effects, competing risks, time-varying covariates, and differential dropout.^11^^–^ ^13^ When the PH assumption does not hold, NMAs that rely on trial-specific HRs may yield biased estimates that result in poor predictions,^14^ which are important for cost-effectiveness modeling.

Several methods have been proposed as alternatives to NMAs based on reported HRs, which have been reviewed by Cope et al.^14^ Crowther et al.^15^ proposed a model for individual patient data (IPD) meta-analysis using Poisson regression models that allows for non-proportional hazards by prespecifying specific time-points at which the HR between two treatments is allowed to change. Freeman and Carpenter^16^ proposed NMA models using cubic splines to model the log-cumulative hazard functions. While flexible, these cubic spline models are restricted to HRs that specifically vary with log-time. Another possibility, proposed by Petit et al.^17^ is to consider the difference in restricted mean survival time at a prespecified time horizon as an alternative to the HR and to fit a standard univariate NMA. While practical, prespecifying the time horizon of interest in accordance with clinical interest may be challenging.

Ouwens et al.^18^ and Jansen^19^ proposed using multivariate parametric NMA models that allow for the HR to change over time. A single parametric survival distribution is used to model interventions in each trial with multivariate relative treatment effects, which are pooled and indirectly compared across trials. This means that interventions can impact both the shape and scale of a selected distribution, resulting in a time-varying treatment effect, which improves model fit and predictions. However, these models were proposed prior to the development of an algorithm to reconstruct event and censor times from the published Kaplan–Meier (KM) curves.^20^ Consequently, they derived discrete hazards from “binned” events over time assuming a binomial likelihood per interval, which may be less accurate than assuming the actual event and censor times follow the likelihood for a particular distribution. Moreover, parameters for these models were transformed to a linear scale, and as a consequence, implementation of these models can be challenging given that the parameterization does not align with standard software (i.e., flexsurv in R).^21^

To address these limitations, Cope et al.^22^ proposed a two-step NMA approach, first fitting parametric survival distributions using frequentist maximum likelihood estimation to each arm of each trial (using the flexsurv R package^23^). In the second step, the arm-specific parameter estimates, their standard errors, and the correlation between the parameters were synthesized using a Bayesian multivariate Normal NMA.^24^

Burke et al.^25^ and Debray et al.^26^ discuss the pros and cons of two-step approaches for IPD meta-analysis relative to one-step approaches. Specifically, the two-step approach for NMA by Cope et al.^22^ offers practical benefits in terms of using established statistical software packages and possibly improving computational burden.^14^^,^ ^27^ However, it was only applied to a subset of relevant standard distributions (Weibull, Gompertz, log-normal, and log-logistic), with parameters transformed to be on a linear scale as in Ouwens et al.^18^ It also relies on bootstrapping to account for parameter correlation^26^^,^ ^28^ and requires assumptions regarding within-study normality and variance.^25^^,^ ^29^ Given that these assumptions can result in certain unexpected biases, Jackson and White^29^ recommend that, when possible, to use one-step methods that make fewer normality assumptions. Therefore, there is a need for one-step NMA models with time-varying treatment effects that can also be applied to distributions such as gamma and generalized gamma that relate the available IPD (whether available or reconstructed) directly to the likelihoods of interest without complex reparameterizations.

In this article, a one-step parametric IPD-NMA framework for TTE outcomes is proposed, which allows for time-varying (i.e., multivariate) treatment effects with the exact likelihood. In Section 2, we extend existing one-step PH models (exponential, Weibull, and Gompertz) or accelerated failure time models (Weibull, log-logistic, gamma, log-normal, and generalized gamma)^27^^,^ ^30^^,^ ^31^ to have time-varying treatment effects. In Section 3, we evaluate the proposed method in a simulation study, and in Section 4, we apply the model to a network of RCTs evaluating multiple interventions for advanced melanoma regarding OS. We conclude in Section 5 with a general discussion, where we explain that proposed models provide the foundation for extensions to the multilevel network meta-regression (ML-NMR) framework to synthesize IPD and aggregate data (AD), while adjusting for potential prognostic factors and effect modifiers.^31^

## 2 A parametric IPD-NMA model for TTE outcomes

We follow the parametrization used by Phillippo^31^ and adopt the reference treatment parameterization defining 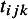 and 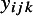 as the event time and censoring indicator, respectively, of individual *i*, in study *j*, with treatment *k*, for *i* in 1,…,*N*, for *j* in 1,…,*J*, and for *k* in 1,…,*K*. Importantly, *k =* 1 is considered the “reference treatment.” We outline the logic and formulas for the Weibull, which can be extended to other standard distributions.

Phillippo^31^ defines a PH Weibull likelihood for IPD (without covariates) as(1)

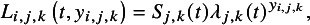

 where the hazard function is(2)

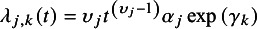

 and the survivor function is(3)

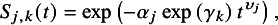

 where 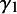 = 0, as treatment 1 is the “reference treatment.” As such, each study has a unique baseline hazard function ( 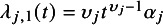 ) defined by the shape parameter, 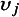 , and scale parameter, 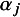 , for study *j*, in 1,…,*J*. This specification restricts the scale and shape parameters to 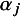 > 0 and 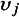 > 0, for *j* in 1,…,*J*, and for *k* in 1,…,*K*. It is noteworthy that when the shape parameter, 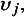 is <1, the hazard rate decreases over time, whereas when 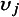 is >1, the hazard rate increases over time. When 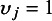 , the hazard rate is constant over time, and the Weibull reduces to the exponential distribution.

The treatment effect is represented by 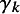 , and Phillippo^31^ proposes that priors for the model parameters can be specified as(4)

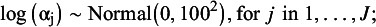


(5)

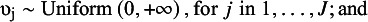


(6)

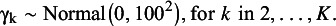



We extend the PH Weibull model proposed by Phillippo^31^ by defining the hazard function as:(7)

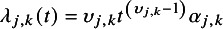

 and the survivor function as(8)

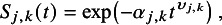

 where 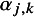 is the scale parameter and 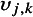 is the shape parameter for the *k*-th treatment in the *j*-th study. An NMA model with an arm-based likelihood^31^ is then specified as(9)

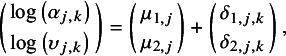

 where 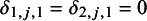 , for *j* in 1,…,*J*. It is noteworthy that, in this notation, the first subscript used for 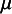 and 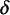 differentiates between the shape and scale parameters. Also note that the PH Weibull model from Phillippo^31^ can be recovered by setting: 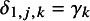 , 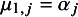 , 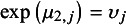 , and 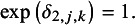


A fixed-effect (FE) NMA model sets 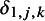 = 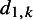 and 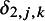 = 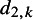 , for *k* in 1,…,*K*, so that treatment effects are not study-specific. Alternatively, a random-effects (RE) NMA model is specified with study-specific treatment effects such that, for *j* in 1,…,*J*, we define the following multivariate normal distribution:(10)

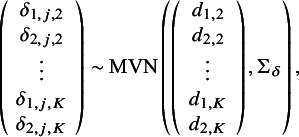

 where 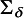 , the 2(*K−*1) by 2(*K−*1) covariance matrix (following Cope et al.^22^), is defined as(11)

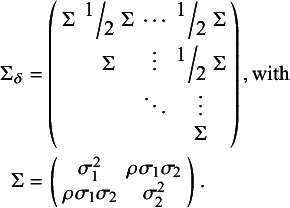



For both the FE and RE models, the 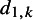 and 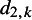 parameters represent the relative effect of treatment *k* versus treatment 1 within each of the *J* study populations (see White et al.^32^).

All Bayesian models require defining priors for all model parameters. For *j* in 1,…,*J*, and for *k* in 2,…,*K*, wide Normal priors with a mean of 0 and a variance of 1,000 can be specified for 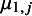 , 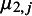 and for 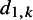 and 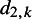 (also following Cope et al.^22^):

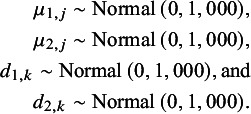



In the RE NMA, a prior is also needed for 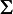 (or alternatively for the individual 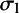 , 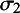 , and 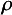 parameters). Following Cope et al.^22^ and Jansen ^19^ we can define the following Inverse-Wishart prior:

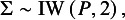

 where *P* is a given 2 by 2 scale matrix.

It is noteworthy that, alternatively, a somewhat less complex RE model could be defined whereby RE are specified for only one of the two treatment effect parameters. This simpler model could be useful when there are reasons to believe that one parameter varies between studies while the rest remain relatively constant. For example, a model could define study-specific treatment effects with respect to the scale parameter, while the shape parameter would remain fixed across studies. For instance, one could define, for *k* in 1, …,*K*, 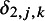 = 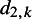 , and for *j* in 1,…, *J*:(12)

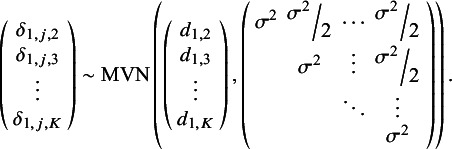



We also note that alternative prior specifications are also possible, and various parameterizations of the variance–covariance matrix specified in equations (11) and (12) could be considered (see Wei and Higgins^33^). Our multivariate Weibull NMA model can be easily generalized to other parametric distributions. We outline standard parametric models in Table 1, including the Weibull, Gompertz, log-normal, log-logistic, gamma, and generalized gamma distributions. The generalized gamma distribution is characterized by three parameters and in our NMA model; only two of these three parameters are study- and treatment-specific. The third parameter, *Q*, is assumed to have the same value for all individuals across all studies and treatments within the network.

**Table 1:** Survival and hazard functions for standard parametric survival models

	Parameters		
Survival distribution	Flexsurv	Model	Survival and hazard functions
Weibull (PH parameterization from Flexsurv)	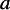	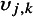	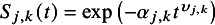
	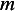	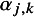	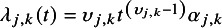
Gompertz	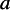	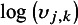	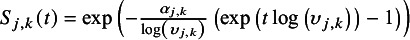
	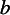	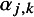	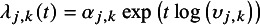
Log-normal	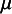	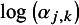	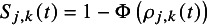
	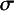	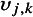	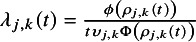
Log-logistic	*b*	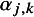	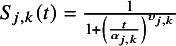
	*a*	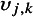	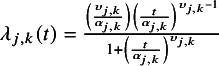
Gamma	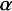	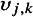	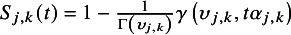
	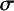	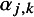	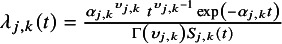
Generalized gamma	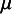	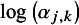	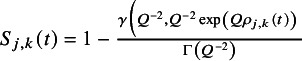 , if 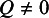 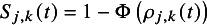 , if 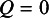
	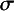	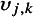	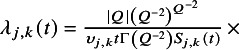 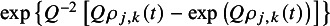
	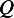	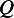	

*Note*: For reference, the parameterization used (“standard parameterization”) corresponds to that used in the flexsurv R package^23^ following the equivalences listed in the “Parameters” columns. Recall that our model specification (equation (9)) restricts the scale and shape parameter to be strictly positive (i.e., 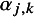 > 0 and 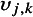 > 0, for j in 1,…,*J*, and for k in 1,…,*K*). Note that 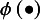 and 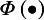 denote the probability density function and the cumulative density function of the standard normal distribution, respectively; 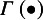 and 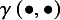 denote the gamma function and the incomplete gamma function, respectively; and 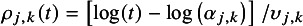 .

## 3 Simulation study

In this section, we consider a simple simulation study to investigate the validity of the proposed one-step model and compare it to the two-step model. In this simulation study, both the proposed one-step model and two-step model from Cope et al.^22^ are used to analyze 5,000 simulated datasets, assuming FE with the Weibull distribution. Jackson and White^29^ suggest that a one-step approach may be preferable due to the “hidden normality” assumptions required with a two-step approach, which can, at least in theory, “have serious implications for the accuracy of the resulting statistical inference.” The simulation study is based on the work in Section 7.3 of Phillippo,^31^ where a single simulated dataset is considered consisting of two RCTs: one trial comparing treatments A versus B, and the other trial comparing treatments A versus C. Here, we extend the network to include a third trial comparing treatments C versus D. In the Supplementary Material, additional analyses are presented to demonstrate how the proposed one-step model fits when different distributions are assumed.

### 3.1 Data-generating mechanism

**Figure 1:**
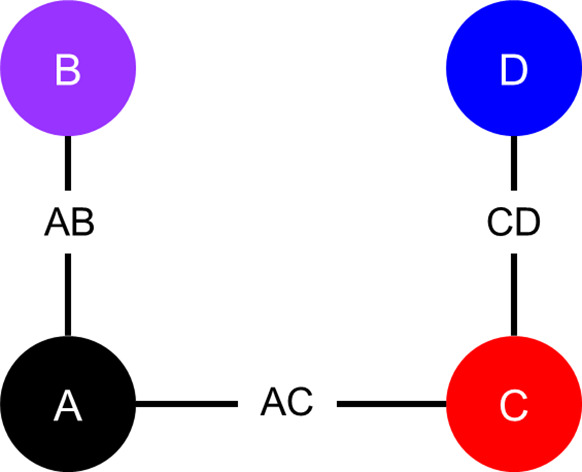
Network diagram of artificial randomized controlled trials.

As illustrated in Figure 1, each artificial dataset includes survival data for three two-arm RCTs: Study *j = 1* compares treatment *k* = 2 to *k* = 1 (henceforth, “Study AB” comparing intervention B with A); Study *j = 2* compares treatment *k* = 3 to *k* = 1 (“Study AC”); and Study *j = 3* compares treatment *k* = 4 to *k* = 3 (“Study CD”). Each study consists of *N_study_* individuals randomly assigned (with a 1:1 randomization ratio) to one of the two treatment arms. We considered two scenarios: (1) with *N_study_* = 36 and (2) with *N_study_* = 100.

We simulated survival times to obtain time-varying treatment effects. Specifically, survival times were simulated from Weibull distributions corresponding to the FE Weibull model outlined in equations (7)–(9) using the cumulative distribution function inversion method (as implemented in the R package simsurv^34^) with the following parameters: 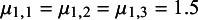 , 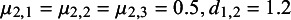 , 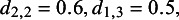 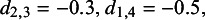 and 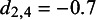 .

**Figure 2:**
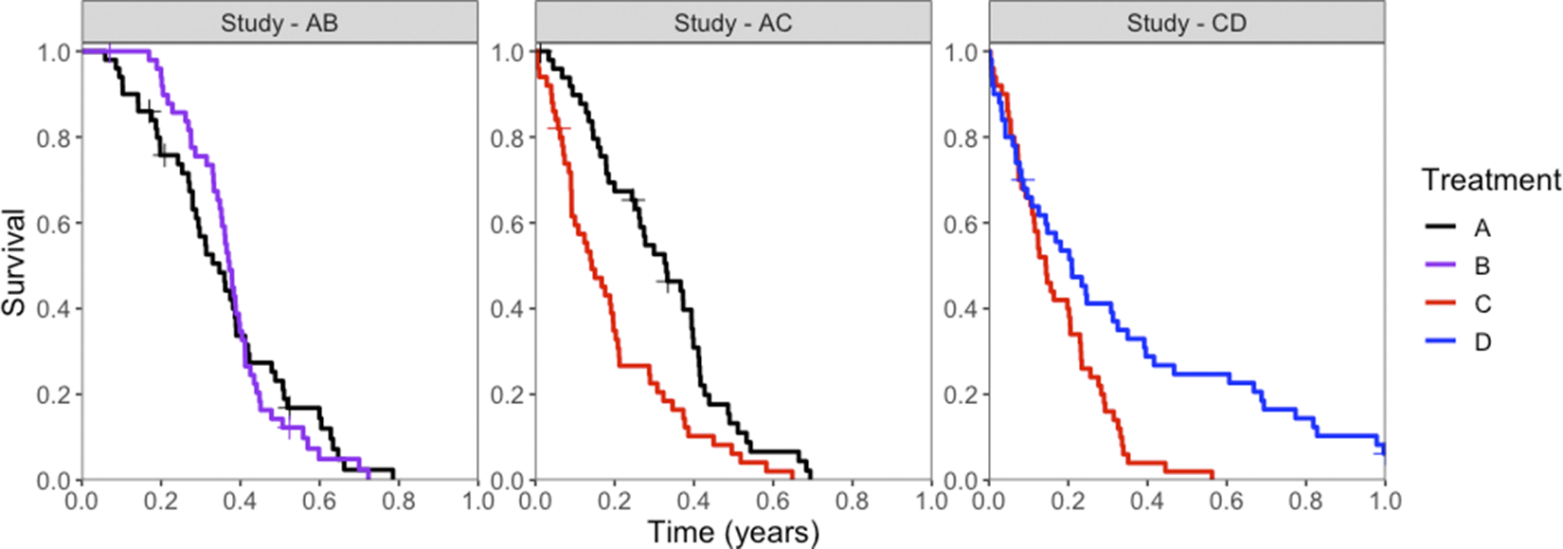
Kaplan–Meier survival curves for simulated event times, for each treatment (colors) in each study (panels). Censored events are marked with a cross (“+”).

Potential censoring times were simulated independently of event times from a Uniform(0,1) distribution, and a random 10% of individuals were selected for potential censoring. An individual was ultimately censored if their survival time was greater than their potential censoring time. All individuals who had not experienced an event by 1 year were censored at 1 year. KM plots illustrate the event and censor times for one of the simulated datasets in Figure 2.

### 3.2 Methods to estimate treatment effects based on the simulated data

The relative treatment effects for the competing interventions based on the artificial RCTs in the network were assessed using a one-step FE IPD-NMA model assuming a Weibull distribution for event times (as outlined in equations (7)–(9)). For comparison, we also fit the data using the two-step approach of Cope et al.,^22^ also assuming an FE model and a Weibull distribution. Both the one-step and two-step models used the parameterization specified in Table 1. The parameters for these models were estimated using the Markov Chain Monte Carlo (MCMC) method using R (packages: rstan, loo, and flexsurv^23^) and Stan,^35^^–^ ^37^ where an initial series of 2,000 iterations from the sampler was discarded (i.e., burn-in) and inferences were based on subsequent 2,000 iterations using four chains.

Results for each dataset were summarized in terms of the posterior medians and 95% credible intervals (CrIs). For each of the two *N_study_* scenarios, across all 5,000 datasets, the mean bias, mean credible interval coverage, and mean credible interval width were calculated.

### 3.3 Results

Results are summarized in Table 2 and suggest that the NMA estimates obtained from both the one-step and two-step models have little to no bias and that both methods provide 95% CrIs with approximately correct coverage (i.e., the 95% CrIs obtained contain the true parameter value ~95% of the time). For almost all parameter estimates, the one-step method appears to be less biased than the two-step method. The average widths of the 95% CrIs are very similar for the one-step and two-step models, suggesting that both approaches are equally efficient. Comparing results from the *N_study_* = 36 scenario with those from the *N_study_* = 100 scenario, we see that with smaller sample sizes (i.e., with *N_study_* = 36), the estimates have larger biases, and the 95% CrIs are perhaps slightly too narrow. Finally, with respect to the computational resources required, the one-step model took about twice as long to fit compared to the two-step model when *N_study_* = 36 and about three times as long to fit when *N_study_* = 100.

**Table 2:** Results from the simulation study: for each parameter, the average estimate (averaged over the 2,000 simulated datasets); the bias (average estimate—truth); 95% CrI coverage (proportion of simulation for which the 95% CrI contained the true parameter value); and average 95% CrI width (averaged over the 5,000 simulated datasets)

			Estimate		Bias		95% CrI coverage		95% CrI width	
			Two-	One-	Two-	One-	Two-	One-	Two-	One-
*N*_study_	Parameter	Truth	step	step	step	step	step	step	step	step
36	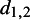	1.20	1.316	1.299	0.116	***0.099***	0.941	0.939	2.524	2.534
	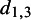	0.50	0.555	0.550	0.055	***0.050***	0.943	0.941	2.131	2.138
	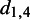	−0.50	−0.544	−0.541	−0.044	***−0.041***	0.932	0.933	2.938	2.949
	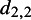	0.60	0.604	0.604	0.004	0.004	0.933	0.942	1.053	1.081
	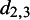	−0.30	−0.289	−0.29	0.011	***0.010***	0.935	0.940	1.053	1.078
	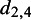	−0.70	−0.703	−0.705	***−0.003***	−0.005	0.935	0.940	1.510	1.543
	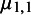	1.50	1.630	1.584	0.130	***0.084***	0.944	0.944	1.348	1.357
	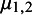	1.50	1.634	1.588	0.134	***0.088***	0.940	0.942	1.353	1.362
	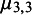	1.50	1.624	1.575	0.124	***0.075***	0.935	0.937	2.692	2.700
	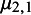	0.50	0.559	0.541	0.059	***0.041***	0.927	0.944	0.749	0.766
	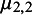	0.50	0.557	0.540	0.057	***0.040***	0.927	0.941	0.750	0.767
	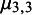	0.50	0.551	0.529	0.051	***0.029***	0.928	0.940	1.286	1.314
100	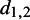	1.20	1.244	1.238	0.044	***0.038***	0.948	0.947	1.421	1.425
	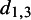	0.50	0.514	0.512	0.014	***0.012***	0.949	0.948	1.205	1.205
	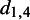	−0.50	−0.522	−0.521	−0.022	***−0.021***	0.943	0.943	1.664	1.665
	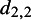	0.60	0.603	0.603	0.003	0.003	0.947	0.946	0.627	0.633
	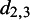	−0.30	−0.301	−0.301	−0.001	−0.001	0.950	0.950	0.627	0.632
	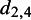	−0.70	−0.705	−0.706	***−0.005***	−0.006	0.950	0.950	0.900	0.906
	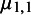	1.50	1.541	1.525	0.041	***0.025***	0.944	0.941	0.772	0.774
	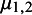	1.50	1.544	1.528	0.044	***0.028***	0.944	0.944	0.775	0.776
	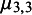	1.50	1.546	1.529	0.046	***0.029***	0.945	0.945	1.517	1.518
	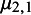	0.50	0.521	0.515	0.021	***0.015***	0.935	0.938	0.446	0.450
	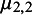	0.50	0.522	0.516	0.022	***0.016***	0.938	0.948	0.448	0.451
	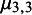	0.50	0.521	0.513	0.021	***0.013***	0.947	0.948	0.766	0.771

*Note*: Survival times were simulated from Weibull distributions corresponding to the FE Weibull model with the following parameters: 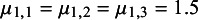 , 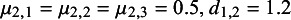 , 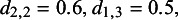 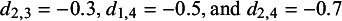 . Bolded values indicate smaller bias value (comparing two-step vs. one-step).

**Figure 3:**
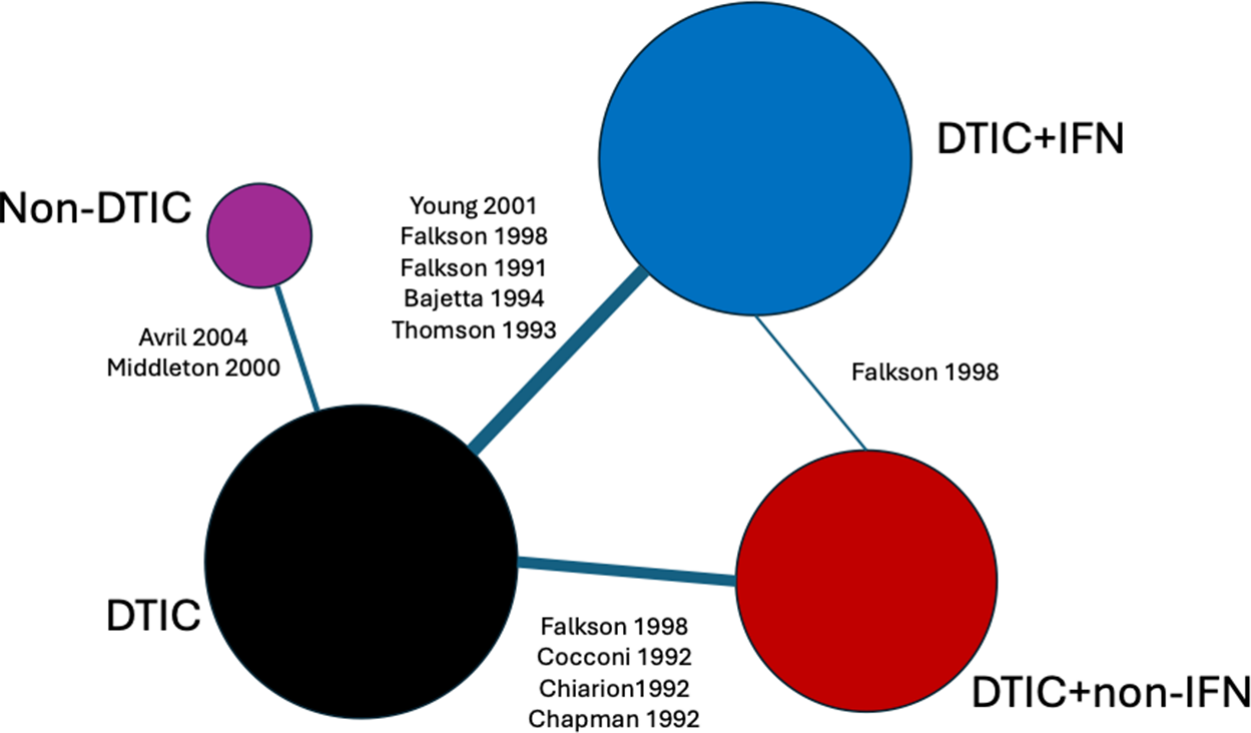
Network of evidence for melanoma randomized controlled trials. Node size and line thickness correspond to the number of studies, including the treatment and the treatment comparison. Abbreviations: DTIC, dacarbazine; IFN, interferon (IFN).

**Figure 4:**
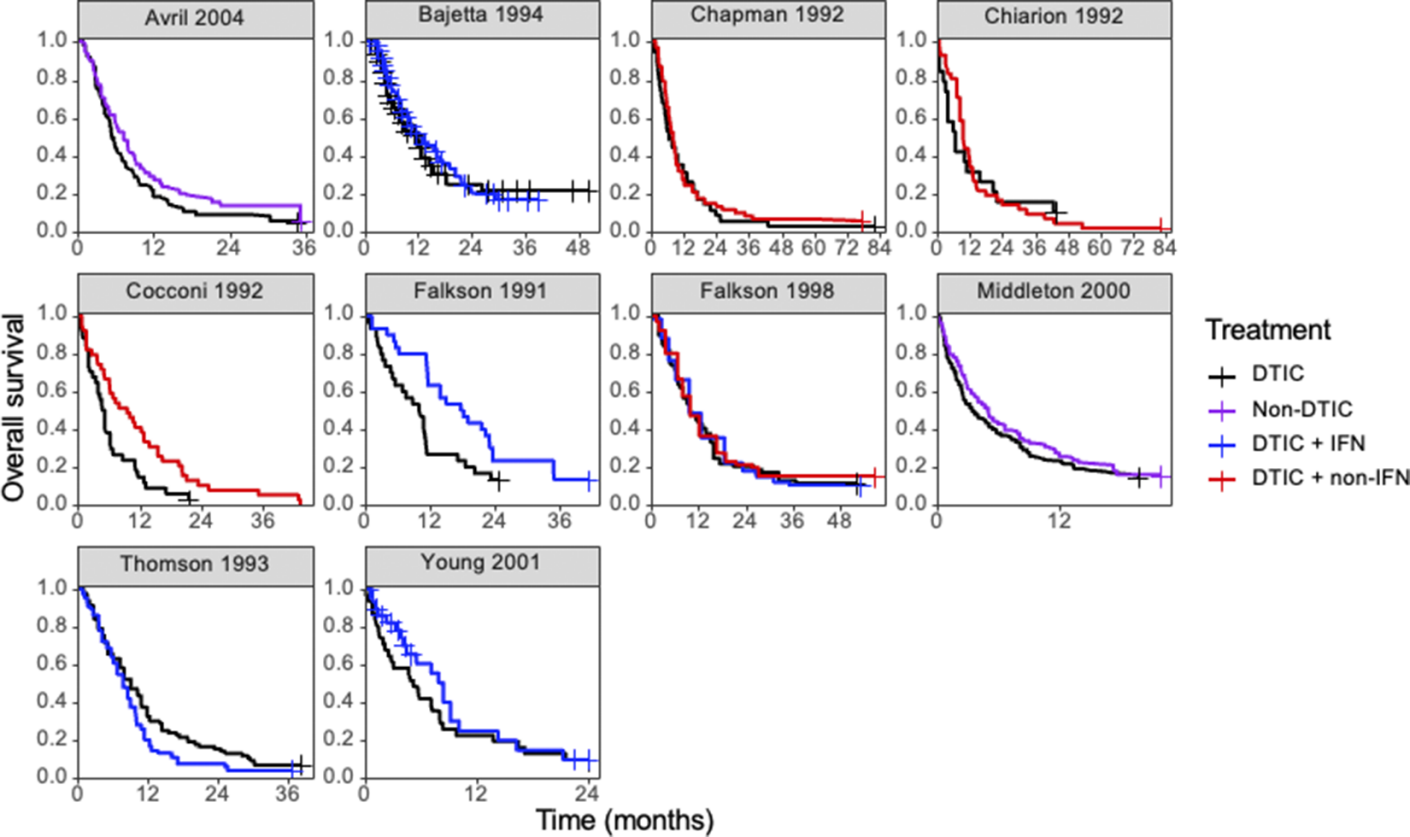
Kaplan–Meier plots of the reconstructed individual event and censoring times obtained for each randomized controlled trial. Abbreviations: DTIC, dacarbazine; IFN, interferon (IFN).

## 4 Illustrative examples with existing empirical data

The illustrative example was based on a network of studies concerning the treatment of advanced (Stage IIIc or IV) melanoma. Details of the network have been presented previously in Jansen et al. ^38^ and Cope et al.^22^ Notably, these studies are somewhat dated, having been published between 1991 and 2004, and we refer readers to Boutros et al.^39^ for a review of the current treatment landscape.

Ten RCTs identified in a systematic literature review formed a connected network, illustrated in Figure 3, evaluating four treatments: dacarbazine (DTIC) monotherapy, DTIC + Interferon (IFN), DTIC + Non-IFN, and Non-DTIC. For each treatment arm in each RCT, the reported KM curves were digitized (DigitizeIt; http://www.digitizeit.de/), and the reconstructed individual event and censoring times were obtained using the Guyot et al.^20^ algorithm (which was recently recommended by Saluja et al.^40^ following an assessment of its reliability, accuracy, and precision) (see Figure 4).

For each of the 10 studies in the melanoma network, Table 3 lists the number of patients per arm, the number of OS events per arm, the median survival time per arm, and the *p*-value obtained from applying the Grambsch and Therneau test for PH.^51^ Notably, there is evidence of non-PH in the Chiarion 1992 study, which compared DTIC and DTIC + non-IFN (*p*-value = 0.021), and therefore, according to Cope et al.^14^ and recent guidance (e.g., “if the PH assumption is deemed to be implausible for one or more comparisons in the network, then (network) meta-analysis of HRs should not be carried out”^52^), a method that does *not* require the PH assumption should be used for analysis.

**Table 3:** For each of the 10 studies in the melanoma network: the number of patients per arm, the number of overall survival events per arm, the median survival time per arm, and the p-value obtained from applying the Grambsch and Therneau test for proportional hazard (PH) assumption

Study	Arm	*N* at risk	Total number of events	Median survival time (months)	*p*-value obtained from the Grambsch and Therneau test for PH
Avril et al.^41^	DTIC	117	111	5.44	0.534
	Non-DTIC	112	105	7.36	
Bajetta et al.^42^	DTIC	82	49	11.80	0.167
	DTIC + IFN	76	48	12.84	
Chapman et al.^43^	DTIC	121	117	6.65	0.359
	DTIC + non-IFN	119	112	8.10	
Chiarion Sileni et al.^44^	DTIC	19	17	6.84	0.021
	DTIC + non-IFN	41	40	9.61	
Cocconi et al.^45^	DTIC	34	33	5.06	0.805
	DTIC + non-IFN	39	39	9.67	
Falkson et al.^46^	DTIC	30	26	10.12	0.063
	DTIC + IFN	30	26	17.85	
Falkson et al.^47^	DTIC	69	61	9.73	0.912
	DTIC + IFN	68	61	9.69	
	DTIC + non-IFN	66	56	9.97	
Middleton et al.^48^	DTIC	149	127	3.43	0.198
	Non-DTIC	156	132	5.04	
Thomson et al.^49^	DTIC	82	76	9.20	0.689
	DTIC + IFN	87	83	7.83	
Young et al.^50^	DTIC	31	28	5.20	0.169
	DTIC + IFN	30	20	8.43	

### 4.1 Methods

We fit the one-step and two-step multivariate NMA models with both the fixed and the REs using MCMC with R and Stan^35^^–^ ^37^ as described in Section 3. We defined REs on both the shape and scale parameters (following equation (10)) and specified an Inverse-Wishart prior for 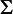 (following equation (11)) with:

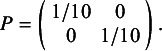



Consistent with Cope et al.,^22^ we considered four different distributions for the likelihood: the Weibull, the Gompertz, the log-normal, and log-logistic. It is noteworthy that, for the two-step approach, the parameterization originally used by Cope et al.^22^ was based on models originally proposed by Ouwens et al.^18^ In contrast, we fit all models with the more familiar parameterization detailed in Table 1.

The approximate leave-one-out (LOO) information criterion (LOOIC) was used to select the most appropriate model. The LOOIC is similar to the DIC in that smaller values signal better model fit, but, unlike the DIC, it is invariant to parametrization and corresponds to a model’s predictive performance (integrating over the posterior distribution of the parameters) (see Vehtari et al.^53^). Convergence for each parameter was assessed using the Gelman-Rubin statistic.^54^^,^ ^55^ Checks for divergent transitions were also performed (see Betancourt et al.^56^).

### 4.2 Results

**Figure 5:**
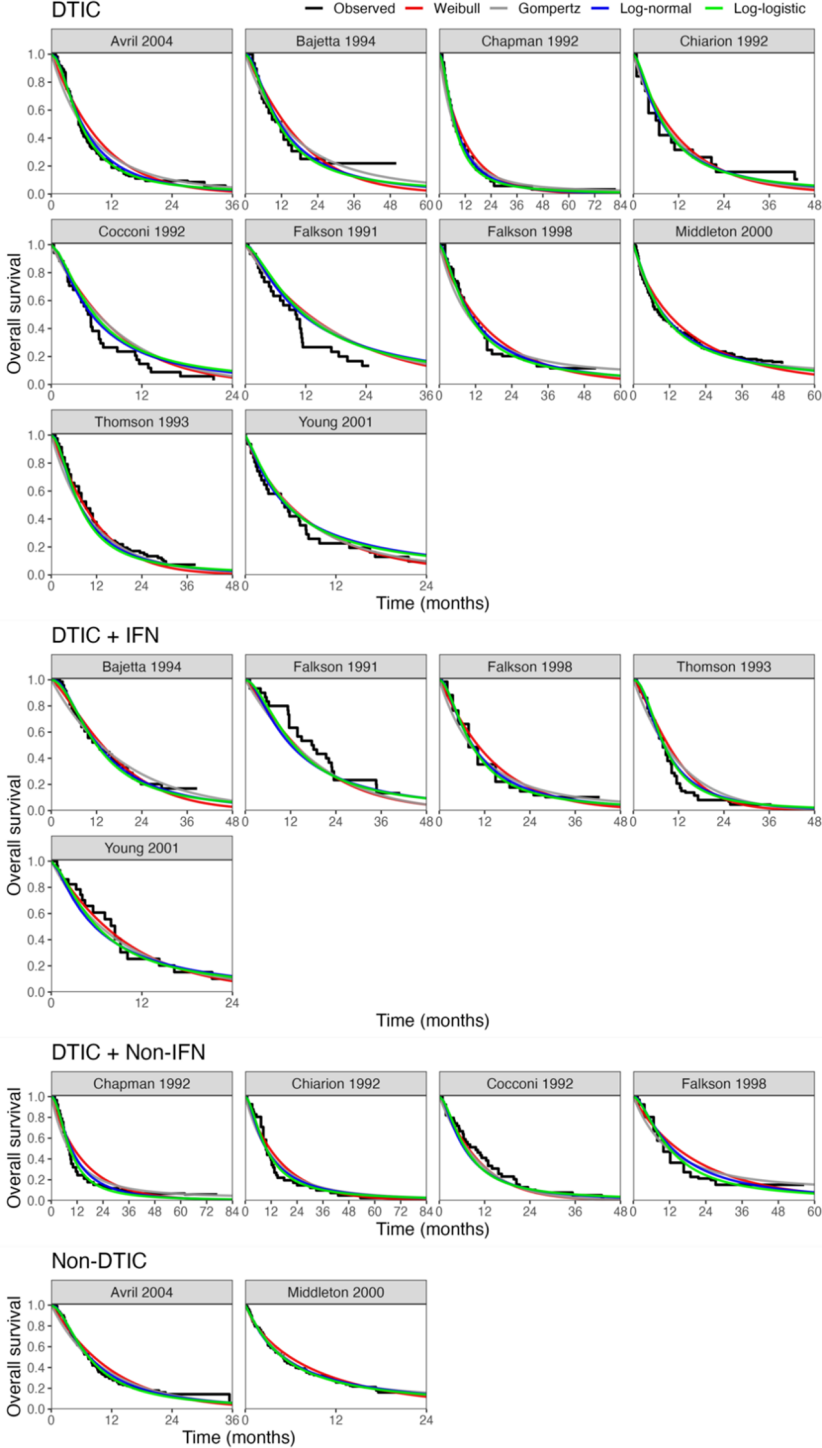
Fitted survival functions for all distributions from FE one-step model (Weibull, Gompertz, log-normal, and log-logistic) and Kaplan–Meier curves by treatment arm. Abbreviations: DTIC, dacarbazine; IFN, interferon (IFN).

**Figure 6:**
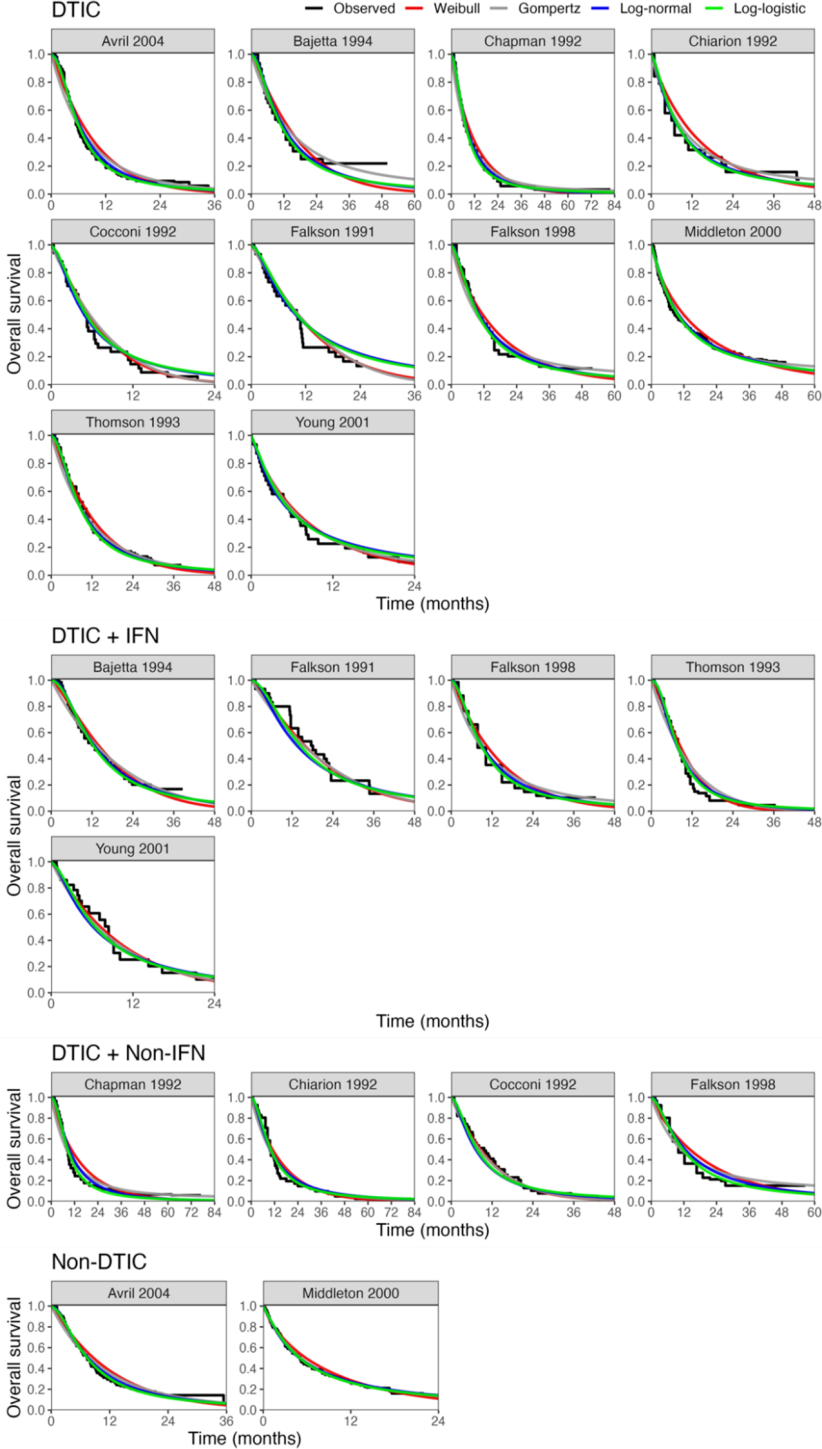
*Fitted study-specific survival functions (using study-specific baseline risk [* 
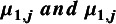
 ] *and relative treatment effects [* 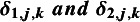 *]) for all distributions from the RE one-step model (Weibull, Gompertz, log-normal, and log-logistic) and Kaplan–Meier curves by treatment arm. Abbreviations: DTIC, dacarbazine; IFN, interferon (IFN).*

**Figure 7:**
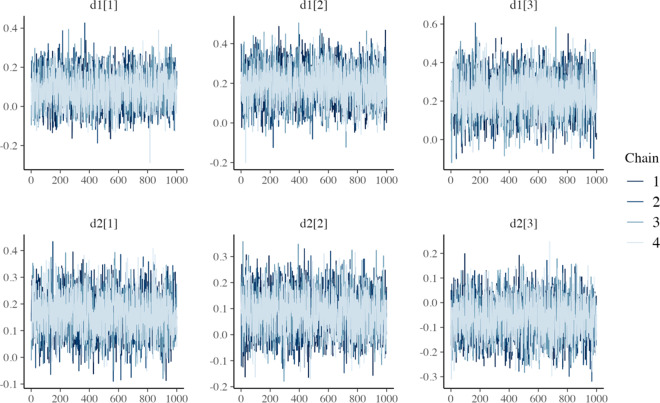
MCMC trace plots for relative treatment effect parameters of the one-step FE log-logistic NMA model.

**Figure 8:**
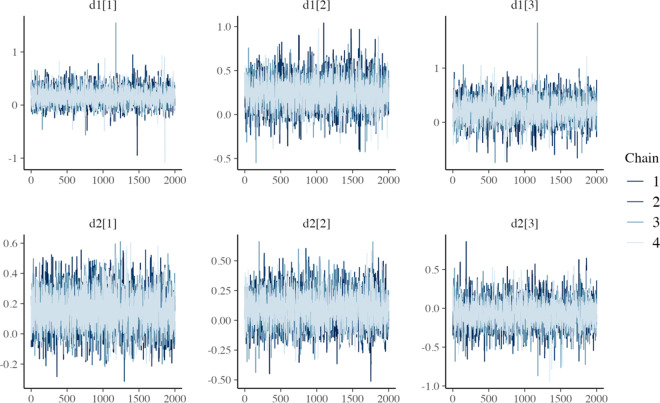
MCMC trace plots for relative treatment effect parameters of the one-step RE log-logistic NMA model.

All models converged and appeared to fit the data reasonably well (see predicted survival curves in Figures 5 and 6, and MCMC trace plots for the log-logistic models in Figures 7 and 8). However, for all models to successfully converge, the RStan default settings for the “adapt_delta” and “max_treedepth” control variables needed to be changed following current recommendations (see details in code provided in the Supplemental Materials).^37^ On a laptop computer (Macbook Pro, with M1 chip and 16 GB memory), the two-step models all took less than a minute to fit, whereas the one-step models took between 1 and 188 minutes (with RE models taking about two and a half times longer to fit than FE models; see Table 4).

The LOOIC suggests that the log-logistic distribution was most appropriate (Table 4), which is consistent with findings by Cope et al.^22^ based on the sum of treatment-arm specific AIC values per distribution in the first step of their analysis. The parameter estimates and 95% CrIs for the one-step log-logistic random-effects (and fixed-effect) model are very similar to those obtained using the two-step NMA method (Table 5). The small discrepancies between the estimates may be explained by the assumptions required in the two-step NMA regarding within-study estimates in terms of normality and standard errors and/or by the different priors required for the one-step and two-step models. These findings were consistent across the alternative distributions for the likelihood evaluated (Table 6), which are summarized in terms of the predicted survivals in Figures 5 and 6.

**Figure 9:**
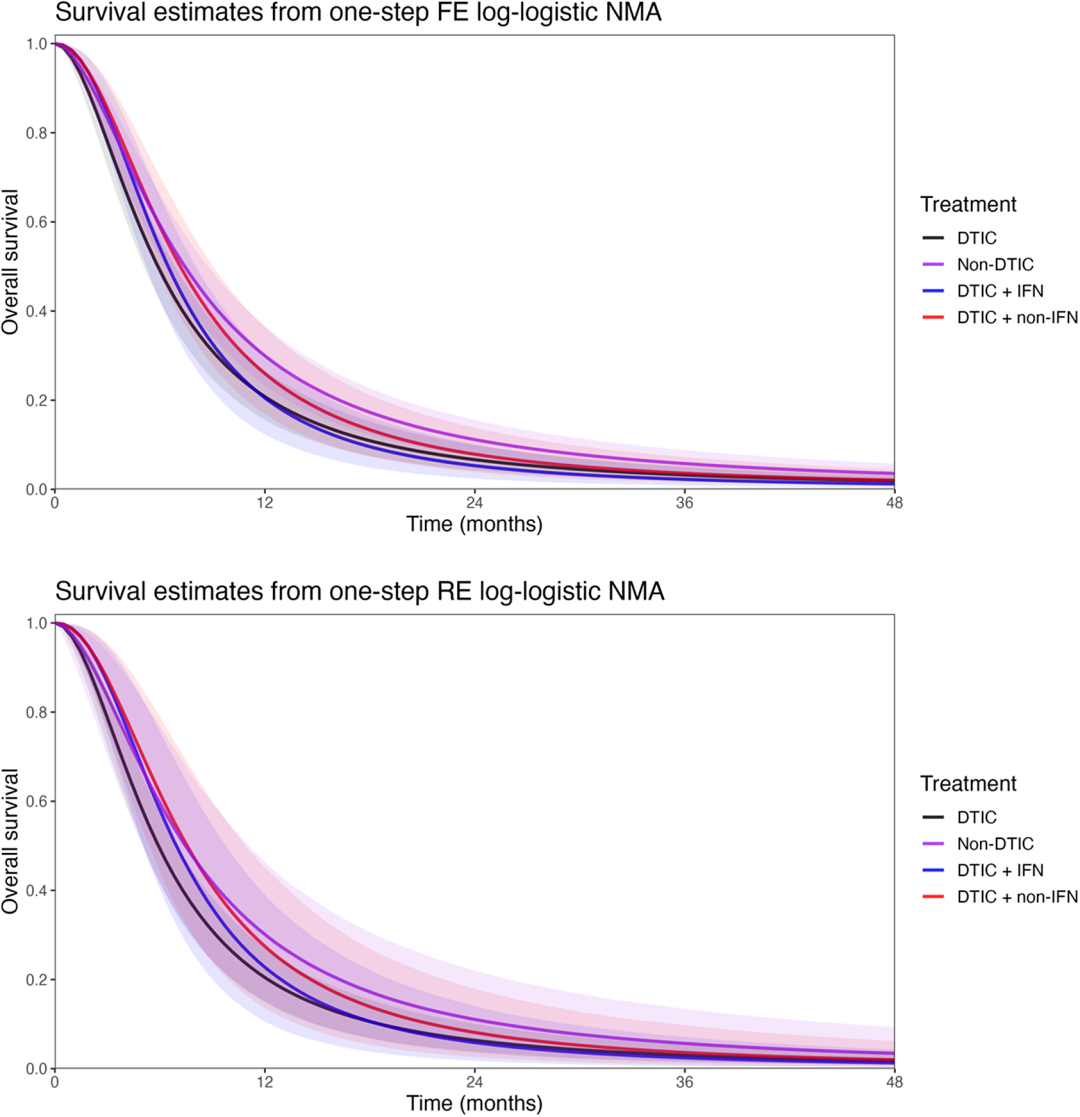
Posterior estimates of overall survival (with shaded 95% CrIs) comparing DTIC, DTIC + IFN, DTIC + non-IFN, and Non-DTIC in the Avril 2004 population from the one-step NMA FE (top panel) and RE (bottom panel) models. Abbreviations: DTIC, dacarbazine; IFN, interferon (IFN).

Figure 9 plots the estimated survival curves from the FE and RE one-step models fit to a population with the same baseline risk as the Avril 2004 study population. Within the first 6 months, survival appears highest for DTIC + non-IFN. However, the DTIC + non-IFN and non-DTIC survival curves cross shortly after 6 months (illustrating the effect of time-varying treatment effects), and among all four treatments, long-term survival appears highest for non-DTIC.

**Table 4:** Computational sampling time required for each model and leave-one-out information criterion (LOOIC)

	Computational sampling time (minutes)	LOOIC	SE of LOOIC
RE model			
Log-logistic	3	9357.51	77.32
Log-normal	2	9365.48	76.28
Gompertz	188	9560.78	75.69
Weibull	136	9628.08	70.45
FE model			
Log-logistic	1	9353.32	77.55
Log-normal	1	9358.66	76.44
Gompertz	67	9549.57	75.69
Weibull	53	9623.45	70.46

*Note*: The LOOIC can be used to select the most appropriate model. The distribution with the lowest LOOIC values, the log-logistic in this case (for both random effects [REs] and fixed effect [FE]), is considered the “best” model. Abbreviation: SE, standard error.

**Table 5:** Parameter estimates (posterior medians and 95% CrIs) obtained with random-effects (REs) and fixed-effect (FE) log-logistic NMA models: (1) two-step multivariate network meta-analysis model (Cope et al.^22^) versus (2) the proposed one-step IPD NMA

Parameter	2-step log-logistic	1-step log-logistic
RE model		
DTIC + IFN ( 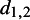 )	0.171 (−0.093, 0.477)	0.158 (−0.113, 0.459)
DTIC + non-IFN ( 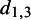 )	0.251 (−0.048, 0.581)	0.243 (−0.060, 0.567)
Non-DTIC ( 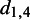 )	0.239 (−0.185, 0.639)	0.233 (−0.177, 0.627)
DTIC + IFN ( 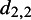 )	0.159 (−0.062, 0.377)	0.160 (−0.071, 0.385)
DTIC + non-IFN ( 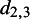 )	0.092 (−0.146, 0.342)	0.095 (−0.145, 0.350)
Non-DTIC ( 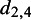 )	−0.067 (−0.379, 0.246)	−0.066 (−0.373, 0.253)
FE model		
DTIC + IFN ( 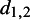 )	0.096 (−0.065, 0.252)	0.089 (−0.075, 0.248)
DTIC + non-IFN ( 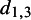 )	0.197 (0.024, 0.371)	0.192 (0.021, 0.364)
Non-DTIC ( 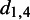 )	0.231 (0.034, 0.424)	0.232 (0.036, 0.432)
DTIC + IFN ( 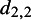 )	0.157 (0.014, 0.299)	0.161 (0.018, 0.307)
DTIC + non-IFN ( 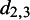 )	0.081 (−0.067, 0.230)	0.086 (−0.063, 0.232)
Non-DTIC ( 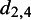 )	−0.061 (−0.208, 0.086)	−0.062 (−0.214, 0.087)

**Table 6:** Parameter estimates (posterior medians and 95% CrIs) obtained with random-effects (REs) and fixed-effect (FE) NMA models: (1) two-step multivariate network meta-analysis model (Cope et al.^22^) versus (2) the proposed one-step IPD NMA

	Weibull	Weibull	Gompertz	Gompertz	Log-normal	Log-normal
Parameter	2-step	1-step	2-step	1-step	2-step	1-step
RE model						
DTIC + IFN ( 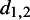 )	−0.475 (−1.006, 0.083)	−0.472 (−1.018, 0.054)	−0.240 (−0.597, 0.102)	−0.235 (−0.593, 0.108)	0.139 (−0.114, 0.436)	0.135 (−0.138, 0.440)
DTIC + non-IFN ( 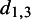 )	−0.264 (−0.809, 0.240)	−0.270 (−0.788, 0.231)	−0.198 (−0.575, 0.153)	−0.192 (−0.588, 0.165)	0.269 (−0.036, 0.605)	0.264 (−0.039, 0.584)
Non-DTIC ( 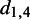 )	−0.185 (−0.716, 0.359)	−0.188 (−0.705, 0.345)	−0.287 (−0.702, 0.154)	−0.282 (−0.732, 0.148)	0.202 (−0.217, 0.617)	0.217 (−0.192, 0.627)
DTIC + IFN ( 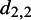 )	0.123 (−0.078, 0.324)	0.124 (−0.078, 0.327)	0.016 (−0.092, 0.127)	0.017 (−0.098, 0.128)	−0.140 (−0.349, 0.065)	−0.141 (−0.349, 0.068)
DTIC + non-IFN ( 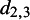 )	0.004 (−0.201, 0.218)	0.009 (−0.199, 0.232)	−0.003 (−0.124, 0.119)	−0.002 (−0.128, 0.128)	−0.064 (−0.296, 0.152)	−0.063 (−0.292, 0.166)
Non-DTIC ( 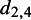 )	−0.008 (−0.270, 0.258)	−0.004 (−0.275, 0.267)	0.017 (−0.164, 0.196)	0.016 (−0.161, 0.201)	0.075 (−0.206, 0.356)	0.070 (−0.215, 0.364)
FE model						
DTIC + IFN ( 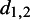 )	−0.412 (−0.888, 0.062)	−0.469 (−0.933, −0.004)	−0.171 (−0.421, 0.084)	−0.175 (−0.429, 0.076)	0.073 (−0.091, 0.230)	0.067 (−0.099, 0.235)
DTIC + non-IFN ( 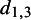 )	−0.235 (−0.659, 0.177)	−0.257 (−0.684, 0.172)	−0.172 (−0.409, 0.069)	−0.173 (−0.406, 0.063)	0.224 (0.055, 0.401)	0.223 (0.046, 0.398)
Non-DTIC ( 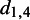 )	−0.163 (−0.519, 0.198)	−0.158 (−0.525, 0.202)	−0.240 (−0.499, 0.013)	−0.244 (−0.500, 0.004)	0.212 (0.020, 0.403)	0.215 (0.015, 0.411)
DTIC + IFN ( 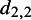 )	0.129 (−0.003, 0.262)	0.146 (0.007, 0.281)	0.015 (−0.004, 0.034)	0.016 (−0.004, 0.035)	−0.134 (−0.265, −0.009)	−0.137 (−0.265, −0.007)
DTIC + non-IFN ( 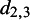 )	0.002 (−0.130, 0.133)	0.013 (−0.122, 0.144)	−0.001 (−0.017, 0.015)	−0.000 (−0.016, 0.015)	−0.049 (−0.174, 0.078)	−0.052 (−0.176, 0.077)
Non-DTIC ( 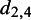 )	−0.022 (−0.153, 0.111)	−0.021 (−0.156, 0.113)	0.009 (−0.017, 0.035)	0.010 (−0.017, 0.037)	0.071 (−0.060, 0.203)	0.070 (−0.058, 0.201)

**Table 7:** Estimates obtained from the one-step and two-step log-logistic models regarding overall survival at 1 year and at 2 years comparing DTIC + IFN and non-DTIC fit for a population with baseline risk of the Avril 2004 study population

	DTIC + IFN (*k* = 2)		Non-DTIC (*k* = 4)		DTIC + IFN versus non-DTIC	
	Two-step	One-step	Two-step	One-step	Two-step	One-step
FE log-logistic with Avril 2004 population						
Overall survival at 1 year (%)	20.49 [12.38, 30.23]	20.29 [12.16, 30.28]	29.96 [23.37, 36.61]	30.05 [23.82, 36.94]	−9.36 [−19.06, 1.45]	−9.65 [−19.79, 0.99]
Overall survival at 2 years (%)	5.29 [2.42, 10.05]	5.28 [2.36, 10.04]	11.15 [7.42, 15.54]	11.27 [7.73, 15.79]	−5.74 [−10.71, −0.51]	−5.90 [−10.87, −0.68]
RE log-logistic with Avril 2004 population						
Overall survival at 1 year (%)	23.19 [11.26, 39.65]	22.73 [10.61, 38.99]	30.10 [14.76, 46.91]	30.12 [15.17, 46.38]	−6.96 [−26.72, 14.22]	−7.16 [−26.56, 14.95]
Overall survival at 2 years (%)	5.93 [1.89, 13.93]	5.86 [1.77, 13.98]	11.02 [3.68, 21.91]	11.04 [3.59, 21.97]	−4.88 [−16.39, 5.25]	−4.97 [−16.28, 5.27]

The treatment effect estimates can be summarized in many ways, and Cope and Jansen^57^ consider how various quantitative summaries compare using the same melanoma network as an illustrative example.^57^ Suppose one was specifically interested in OS at 1 year and at 2 years, and was interested in comparing DTIC + IFN and non-DTIC, a treatment comparison for which there is no direct evidence. Table 7 lists the relevant estimates obtained from the one-step and two-step log-logistic models fit to a population with the same baseline risk as the Avril 2004 study population. Briefly, with the one-step FE log-logistic model, the OS at 1 year with DTIC + IFN is estimated to be 20% (95% CrI: [12%, 30%]), slightly lower than with non-DTIC: 30% (95% CrI: [24%, 37%]). The estimated difference in OS at 1 year is −10% (95% CrI: [−20%, 1%]), and at 2 years is −6% (95% CrI: [−11%, −1%]). These findings were consistent for one-step and two-step models. Credible intervals are notably wider with RE models relative to FE models.

## 5 Discussion

The proposed multivariate NMA models for TTE outcomes provide a one-step framework that allows for time-varying treatment effects, which leverage exact (or reconstructed) event and censor times for the studies in a connected network of evidence. By using the exact likelihood specification, we avoid the assumptions regarding within-study normality and variance required in the two-step method (Cope et al.^22^). This allows the entire model to be fit within a Bayesian framework, facilitating straightforward model selection and interpretation. Potential concerns regarding computational burden are mitigated by using Stan software, which provides a more efficient MCMC sampling than previous software (i.e., WinBUGS or JAGS) through the implementation of the Hamiltonian Monte Carlo algorithm (see Monnahan et al.^58^).

While we discussed using the LOOIC for model selection, often, valuable insight regarding the plausibility of different models may also be obtained from clinical experts^59^^–^ ^61^ and observational studies.^62^ Moreover, when there is no reason to believe that treatment effects vary over time, simpler models with fewer parameters may be more appropriate. Cope et al.^14^ previously proposed a stepwise process exploring standard parametric models with treatment effect on scale alone, followed by models with multivariate treatment effects (scale and shape) for each trial and network (or other more flexible models), which builds upon the process outlined by Latimer et al.^59^ for survival analysis of a single RCT in context of cost-effectiveness analysis. The proposed one-step models allow this process to be applied to all the “standard” distributions, now including gamma and generalized gamma. Further, the generalized gamma distribution may be particularly useful to improve fit over other standard distributions to a network of RCTs given the flexibility to model U-shaped hazards with this distribution^63^ as well as the nature of its nested distributions (i.e., exponential, Weibull, log-normal, and gamma).

The simulation study in Section 3 found that the proposed one-step method and the previously proposed two-step method provide broadly similar results. When study sample sizes are especially small, the one-step method may be preferred, in line with previous recommendations.^29^ However, due to finite computational resources, the simulation study was limited in that we were unable to consider fitting RE models and did not consider a wide range of sample sizes and prior specifications. More extensive simulation studies may be helpful to better understand the impact of model misspecification, the differences between fitting FE and RE models, and the differences between using one-step and two-step methods.^64^

The illustrative melanoma example highlighted an application to a network of multiple trials, where we demonstrated the feasibility of accounting for between-study heterogeneity in terms of both the shape and the scale parameters. However, simpler random-effect models specified for only one of the two treatment effect parameters may often be sufficient. We advise consulting clinicians to consider whether between-study heterogeneity is most likely to affect the scale versus the shape of the distributions. Future research should consider the merits of using different priors and parameterizations for the variance–covariance matrix,^65^^,^ ^66^ potentially based on incorporating external evidence on the between-trial heterogeneity.^67^

It may be of interest to extend the proposed methods to nonparametric IPD-NMA models. While potentially flexible, nonparametric models can be considerably more complex and may not be among the first NMA models to consider when following recommended model selection procedures in the broader cost-effectiveness framework.^59^ Nonetheless, there may be utility in extending the current framework to include additional nonparametric methods.

During the development of this publication, Phillippo et al. proposed the extension the ML-NMR framework for TTE data, considering networks in which IPD-level covariate data is only available for a subset of trials within the network (with aggregate-level covariate data available for the remaining studies).^68^ This allows for the adjustment of prognostic factors and effect modifiers when information on these is available. Maciel et al.^69^ demonstrated the feasibility of applying ML-NMR for TTE outcomes with a univariate treatment effect. The main advantage of NMA models with a multivariate treatment effect as proposed is that they do not rely on a PH assumption across studies and treatment comparisons. This flexibility may be important when there are differences in the survival distributions for the treatments compared, and relative treatment effects need to be extrapolated beyond the available trial data for a cost-effectiveness analysis.

## Supporting information

Campbell et al. supplementary material Campbell et al. supplementary material

## Data Availability

We provide Stan code for both the two-step and one-step FE and RE models in the Supplementary Material. Also in the Supplementary Material, R code and data are provided to fit the one-step NMA models to the illustrative melanoma example from Section 4.

## Supplementary material

To view supplementary material for this article, please visit http://doi.org/10.1017/rsm.2025.21.
