# Supplementary material for: One-step parametric network meta-analysis models using the exact likelihood that allow for time-varying treatment effects
Source: Res Synth Methods. 2025 May 15;16(4):650–71. doi: 10.1017/rsm.2025.21 (PMC12527511; doi:10.1017/rsm.2025.21)
Supplement: Campbell et al. supplementary material [file S1759287925000213sup001.zip › Supplementary Appendix.docx]

# **Appendix**

# Additional analyses with an artificial illustrative example with simulated data

In order to investigate how the proposed one-step NMA model fit when assuming distributions other than the Weibull, we simulated a single dataset following the same procedure used in the Section 3 simulation study, but with larger sample sizes of 1000 individuals per study. KM plots illustrate the event and censor times in **Figure A1**.

The relative treatment effects for the competing interventions based on this artificial dataset were assessed using a fixed-effect one-step IPD-NMA model assuming either the Weibull, Gompertz, log-normal, log-logistic, gamma, or generalized gamma (as detailed in **Table 1**), all of which assume a time-varying treatment effect. Parameters for these models were estimated with MCMC as in the Simulation study in Section 3. For comparison, we also fit the data using frequentist maximum likelihood estimation applied to the data from each study separately using the flexsurv R package ^23^.

All models converged and results are summarized in terms of the posterior medians and 95% credible intervals (CrIs) are listed in **Table A1.** These results demonstrate that the NMA estimates and 95% CrIs align well with the confidence intervals obtained from frequentist study-level fits using flexsurv. With each of the alternative distributions, the estimates and 95% CrIs obtained align almost exactly with those obtained from study-level fits using flexsurv. This suggests that the linear transformations previously made with the two-step method ^22^ are not necessary. Note that for the generalized gamma distribution, the study-level flexsurv models provide a different estimate of the *Q* parameter for each study, whereas the NMA provides a single overall estimate. The corresponding survival and hazards over time are illustrated for all distributions in **Figure A2** and suggest that these models can provide an unbiased flexible framework to evaluate multivariate treatment effects.

# Stan code for one-step and two-step models

**Two-step FE model:**

data {

// Constants

int<lower = 1> ntrt; // number of treatments

int<lower = 1> ns; // number of studies

int<lower = 1> n_arm; // total number of patients

int<lower = 1> study_id[n_arm]; // total number of patients

int<lower = 0> trt_id[n_arm]; // total number of patients

matrix[n_arm,2] y;

matrix[2,2] omega[n_arm];

// sd for priors on mus and ds (thought: could even place priors on these)

real<lower = 0> mu_prior_sd;

real<lower = 0> d_prior_sd;

}

parameters {

vector[2*ns] mu;

vector[2*(ntrt-1)] d;

}

transformed parameters{

matrix[n_arm,2] meany;

for(a in 1:n_arm) {

if(trt_id[a]>0){

meany[a,1] = mu[2*study_id[a]-1] +

d[(2*trt_id[a]-1)];

meany[a,2] = mu[2*study_id[a]] +

d[(2*trt_id[a])];}

if(trt_id[a]==0){

meany[a,1] = mu[(2*study_id[a]-1)] ;

meany[a,2] = mu[2*study_id[a]];}

}

}

model {

// Priors for study baselines

for(j in 1:ns) {

mu[j] ~ normal(0, mu_prior_sd);

}

// Priors for treatment effects

for(k in 1:(ntrt - 1)){

d[k] ~ normal(0, d_prior_sd);

}

// Likelihood

for(a in 1:n_arm) {

y[a,1:2] ~ multi_normal(meany[a,1:2], omega[a,1:2,1:2]); # multivariate likelihood

}

}

generated quantities {

// split mus, ds, and deltas into those for loc and anc

vector[ns] mu1;

vector[ns] mu2;

for(j in 1:ns) {

mu1[j] = mu[2*j-1];

mu2[j] = mu[2*j];

}

vector[ntrt - 1] d1;

vector[ntrt - 1] d2;

for(k in 1:(ntrt - 1)){

d1[k] = d[2*k-1];

d2[k] = d[2*k];

}

}

**Two-step RE model:**

data {

// Constants

int<lower = 1> ntrt; // number of treatments

int<lower = 1> ns; // number of studies

int<lower = 1> n_arm; // total number of patients

int<lower = 1> study_id[n_arm]; // total number of patients

int<lower = 0> trt_id[n_arm]; // total number of patients

matrix[n_arm,2] y;

matrix[2,2] omega[n_arm];

// sd for priors on mus and ds (thought: could even place priors on these)

real<lower = 0> mu_prior_sd;

real<lower = 0> d_prior_sd;

}

parameters {

vector[2*ns] mu;

vector[2*(ntrt-1)] d;

}

transformed parameters{

matrix[n_arm,2] meany;

for(a in 1:n_arm) {

if(trt_id[a]>0){

meany[a,1] = mu[2*study_id[a]-1] +

d[(2*trt_id[a]-1)];

meany[a,2] = mu[2*study_id[a]] +

d[(2*trt_id[a])];}

if(trt_id[a]==0){

meany[a,1] = mu[(2*study_id[a]-1)] ;

meany[a,2] = mu[2*study_id[a]];}

}

}

model {

// Priors for study baselines

for(j in 1:ns) {

mu[j] ~ normal(0, mu_prior_sd);

}

// Priors for true treatment effects

for(k in 1:(ntrt - 1)){

d[k] ~ normal(0, d_prior_sd);

}

// Likelihood

for(a in 1:n_arm) {

y[a,1:2] ~ multi_normal(meany[a,1:2], omega[a,1:2,1:2]); # multivariate likelihood

}

}

generated quantities {

// split mus, ds, and deltas into those for loc and anc

vector[ns] mu1;

vector[ns] mu2;

for(j in 1:ns) {

mu1[j] = mu[2*j-1];

mu2[j] = mu[2*j];

}

vector[ntrt - 1] d1;

vector[ntrt - 1] d2;

for(k in 1:(ntrt - 1)){

d1[k] = d[2*k-1];

d2[k] = d[2*k];

}

}

**One-step FE model:**

functions {

// Gompertz

real gompertz_lpdf (real y, real scale, real shape){

real log_scale = log(scale);

real numerator = log_scale + shape*y;

return numerator - scale/shape * expm1(shape*y);

}

real gompertz_lccdf (real y, real scale, real shape){

return -scale/shape * expm1(shape*y);

}

// log-logistic (if undefined depending on version of stan)

// real loglogistic_lpdf (real y, real alpha, real beta){

// real numerator = log(beta) - log(alpha) + (beta - 1)*(log(y) - log(alpha));

// return numerator - 2*(log1p((y/alpha)^beta));

// }

real loglogistic_lccdf (real y, real alpha, real beta){

return -log1p( (y / alpha)^beta);

}

// generalized gamma (Lawless parameterization)

real gengamma_lpdf(real y, real mu, real sigma, real Q) {

real k = pow(Q, -2);

real w = Q * (log(y) - mu) / sigma;

return -log(sigma) - log(y) - 0.5 * log(k) * (1 - 2 * k) +

k * (w - exp(w)) - lgamma(k);

}

real gengamma_lccdf(real y, real mu, real sigma, real Q) {

real k = pow(Q, -2);

real w = exp(Q .* (log(y) - mu) / sigma) .* k;

return log1m(gamma_p(k, w));

}

}

data {

// Constants

int<lower = 1> ntrt; // number of treatments

int<lower = 1> ns; // number of studies

int<lower = 1> N; // total number of patients

int<lower = 1, upper = 6> dist; // distribtion flag

//(1 = weibull, 2 = Gompertz, 3 = gamma,

// 4 = log-normal, 5 = log-logistic,

// 6 = generalized gamma)

// Survival data

vector<lower = 0>[N] time; // event & censor times

int<lower = 0, upper = 1> event[N]; // event indicator (0=censored, 1=event)

int<lower = 1> study[N]; // study indicator

// model matrix

matrix[N, ns + ntrt - 1] X;

// sd for priors on mus, ds, and gengamma Q

real<lower = 0> mu_prior_sd;

real<lower = 0> d_prior_sd;

real<lower = 0> Q_prior_sd;

}

parameters {

// location parameter

vector[ns + ntrt - 1] loc;

// ancilliary parameter

vector[ns + ntrt - 1] anc;

// Q for gengamma (one across all studies)

real Q;

}

transformed parameters{

// define mus and ds from loc and anc -> order doesn't matter for FE

vector[2*ns] mu = append_row(loc[1:ns], anc[1:ns]);

vector[2*(ntrt-1)] d = append_row(loc[(ns + 1):(ns + ntrt - 1)],

anc[(ns + 1):(ns + ntrt - 1)]);

// define scale and shapes to be used in likelihoods

vector[N] Loc = X * loc;

vector[N] eLoc = exp(X * loc);

vector[N] Anc = X * anc;

vector[N] eAnc = exp(X * anc);

}

model {

// Priors for study baselines

mu ~ normal(0, mu_prior_sd);

// Priors for treatment effects

d ~ normal(0, d_prior_sd);

// Prior for Q of generalized gamma

Q ~ normal(0, Q_prior_sd);

// Likelihood

if(dist == 1) { // Weibull

for(i in 1:N) {

if(event[i] == 1)

target += weibull_lpdf(time[i] | eAnc[i],

pow(eLoc[i], -1/eAnc[i]));

else

target += weibull_lccdf(time[i] | eAnc[i],

pow(eLoc[i], -1/eAnc[i]));

}

} else if(dist == 2) { // Gompertz (no stan function for this)

for(i in 1:N) {

if(event[i] == 1)

target += gompertz_lpdf(time[i] | eLoc[i], Anc[i]);

else

target += gompertz_lccdf(time[i] | eLoc[i], Anc[i]);

}

} else if(dist == 3) { // Gamma distribution

for(i in 1:N) {

if(event[i] == 1)

target += gamma_lpdf(time[i] | eLoc[i], eAnc[i]);

else

target += gamma_lccdf(time[i] | eLoc[i], eAnc[i]);

}

} else if(dist == 4) { // Log-normal distribution

for(i in 1:N) {

if(event[i] == 1)

target += lognormal_lpdf(time[i] | Loc[i], eAnc[i]);

else

target += lognormal_lccdf(time[i] | Loc[i], eAnc[i]);

}

} else if(dist == 5) { // Log-logistic distribution

for(i in 1:N) {

if(event[i] == 1)

target += loglogistic_lpdf(time[i] | eLoc[i], eAnc[i]);

else

target += loglogistic_lccdf(time[i] | eLoc[i], eAnc[i]);

}

} else if(dist == 6) { // Generalized gamma distribution

for(i in 1:N) {

if(event[i] == 1)

target += gengamma_lpdf(time[i] | Loc[i], eAnc[i], Q);

else

target += gengamma_lccdf(time[i] | Loc[i], eAnc[i], Q);

}

}

}

generated quantities {

// split mus and ds into those for loc and anc

vector[ns] mu1 = loc[1:ns];

vector[ntrt - 1] d1 = loc[(ns + 1):(ns + ntrt - 1)];

vector[ns] mu2 = anc[1:ns];

vector[ntrt - 1] d2 = anc[(ns + 1):(ns + ntrt - 1)];

// log likelihood for use with loo package

vector[N] log_lik;

if(dist == 1) { // Weibull

for(i in 1:N) {

if(event[i] == 1)

log_lik[i] = weibull_lpdf(time[i] | eAnc[i],

pow(eLoc[i], -1/eAnc[i]));

else

log_lik[i] = weibull_lccdf(time[i] | eAnc[i],

pow(eLoc[i], -1/eAnc[i]));

}

} else if(dist == 2) { // Gompertz (no stan function for this)

for(i in 1:N) {

if(event[i] == 1)

log_lik[i] = gompertz_lpdf(time[i] | eLoc[i], Anc[i]);

else

log_lik[i] = gompertz_lccdf(time[i] | eLoc[i], Anc[i]);

}

} else if(dist == 3) { // Gamma distribution

for(i in 1:N) {

if(event[i] == 1)

log_lik[i] = gamma_lpdf(time[i] | eAnc[i], eLoc[i]);

else

log_lik[i] = gamma_lccdf(time[i] | eAnc[i], eLoc[i]);

}

} else if(dist == 4) { // Log-normal distribution

for(i in 1:N) {

if(event[i] == 1)

log_lik[i] = lognormal_lpdf(time[i] | Loc[i], eAnc[i]);

else

log_lik[i] = lognormal_lccdf(time[i] | Loc[i], eAnc[i]);

}

} else if(dist == 5) { // Log-logistic distribution

for(i in 1:N) {

if(event[i] == 1)

log_lik[i] = loglogistic_lpdf(time[i] | eLoc[i], eAnc[i]);

else

log_lik[i] = loglogistic_lccdf(time[i] | eLoc[i], eAnc[i]);

}

} else if(dist == 6) { // Generalized gamma distribution

for(i in 1:N) {

if(event[i] == 1)

log_lik[i] = gengamma_lpdf(time[i] | Loc[i], eAnc[i], Q);

else

log_lik[i] = gengamma_lccdf(time[i] | Loc[i], eAnc[i], Q);

}

}

}

**One-step RE model:**

functions {

// Gompertz

real gompertz_lpdf (real y, real scale, real shape){

real log_scale = log(scale);

real numerator = log_scale + shape*y;

return numerator - scale/shape * expm1(shape*y);

}

real gompertz_lccdf (real y, real scale, real shape){

return -scale/shape * expm1(shape*y);

}

// log-logistic (if undefined depending on version of stan)

// real loglogistic_lpdf (real y, real alpha, real beta){

// real numerator = log(beta) - log(alpha) + (beta - 1)*(log(y) - log(alpha));

// return numerator - 2*(log1p((y/alpha)^beta));

// }

real loglogistic_lccdf (real y, real alpha, real beta){

return -log1p((y / alpha)^beta);

}

// generalized gamma (Lawless parameterization)

real gengamma_lpdf(real y, real mu, real sigma, real Q) {

real k = pow(Q, -2);

real w = Q * (log(y) - mu) / sigma;

return -log(sigma) - log(y) - 0.5 * log(k) * (1 - 2 * k) +

k * (w - exp(w)) - lgamma(k);

}

real gengamma_lccdf(real y, real mu, real sigma, real Q) {

real k = pow(Q, -2);

real w = exp(Q .* (log(y) - mu) / sigma) .* k;

return log1m(gamma_p(k, w));

}

}

data {

// Constants

int<lower = 1> ntrt; // number of treatments

int<lower = 1> ns; // number of studies

int<lower = 1> N; // total number of patients

int<lower = 1, upper = 6> dist; // distribtion flag

//(1 = weibull, 2 = Gompertz, 3 = gamma,

// 4 = log-normal, 5 = log-logistic,

// 6 = gen gamma)

// Survival data

vector<lower = 0>[N] time; // event & censor times

int<lower = 0, upper = 1> event[N]; // event indicator (0=censored, 1=event)

int<lower = 1> study[N]; // study indicator

// model matrix

matrix[N, ns + ns*(ntrt - 1)] X;

// sd for priors on mus, ds, and gengamma Q

real<lower = 0> mu_prior_sd;

real<lower = 0> d_prior_sd;

real<lower = 0> Q_prior_sd;

//sd for prior on within-treatment between parameter covariance matrix

real<lower = 0> sigma_prior_sd;

}

parameters {

// location parameter

real loc_mu[ns];

vector[ntrt - 1] loc_delta[ns];

// ancilliary parameter

real anc_mu[ns];

vector[ntrt - 1] anc_delta[ns];

// true treatment effects;

vector[2*(ntrt-1)] d;

// within-treatment between parameter covariance matrix

cov_matrix[2] sigma;

// Q for gengamma (one across all studies)

real Q;

}

transformed parameters{

// study baselines

vector[2*ns] mu;

for(j in 1:ns){

mu[(2*j-1)] = loc_mu[j];

mu[2*j] = anc_mu[j];

}

// study specific treatment effects

vector[2*(ntrt - 1)] delta[ns];

for(j in 1:ns) {

for(k in 1:(ntrt - 1)) {

delta[j, (2*k - 1)] = loc_delta[j, k];

delta[j, (2*k)] = anc_delta[j, k];

}

}

// within study covariance matrix of the deltas

cov_matrix[2*(ntrt - 1)] delta_sigma;

for(k in 1:(ntrt - 1)) {

for(kk in 1: (ntrt - 1)) {

delta_sigma[(2*k-1):(2*k),(2*kk-1):(2*kk)] = k == kk ? sigma : 0.5*sigma;

}

}

// define linear predictors for location and ancilliary parameters

vector[ns + ns*(ntrt - 1)] loc;

vector[ns + ns*(ntrt - 1)] anc;

loc[1:ns] = to_vector(loc_mu);

anc[1:ns] = to_vector(anc_mu);

for(j in 1:ns) {

loc[(ns+1+(j-1)*(ntrt-1)):(ns+j*(ntrt-1))] = loc_delta[j];

anc[(ns+1+(j-1)*(ntrt-1)):(ns+j*(ntrt-1))] = anc_delta[j];

}

// define location and ancilliary parameters to be used in likelihoods

vector[N] Loc = X * loc;

vector[N] eLoc = exp(X * loc);

vector[N] Anc = X * anc;

vector[N] eAnc = exp(X * anc);

}

model {

// Priors for study baselines

mu ~ normal(0, mu_prior_sd);

// Priors for study-specific treatment effects

for(j in 1:ns) {

delta[j] ~ multi_normal(d, delta_sigma);

}

// Hyperpriors for true treatment effects

d ~ normal(0, d_prior_sd);

// Hyperprior on within study covariance of treatment effects

sigma ~ inv_wishart(2, diag_matrix(rep_vector(pow(sigma_prior_sd,-2),2)));

// Prior for Q of generalized gamma

Q ~ normal(0, Q_prior_sd);

// Likelihood

if(dist == 1) { // Weibull

for(i in 1:N) {

if(event[i] == 1)

target += weibull_lpdf(time[i] | eAnc[i],

pow(eLoc[i], -1/eAnc[i]));

else

target += weibull_lccdf(time[i] | eAnc[i],

pow(eLoc[i], -1/eAnc[i]));

}

} else if(dist == 2) { // Gompertz (no stan function for this)

for(i in 1:N) {

if(event[i] == 1)

target += gompertz_lpdf(time[i] | eLoc[i], Anc[i]);

else

target += gompertz_lccdf(time[i] | eLoc[i], Anc[i]);

}

} else if(dist == 3) { // Gamma distribution

for(i in 1:N) {

if(event[i] == 1)

target += gamma_lpdf(time[i] | eAnc[i], eLoc[i]);

else

target += gamma_lccdf(time[i] | eAnc[i], eLoc[i]);

}

} else if(dist == 4) { // Log-normal distribution

for(i in 1:N) {

if(event[i] == 1)

target += lognormal_lpdf(time[i] | Loc[i], eAnc[i]);

else

target += lognormal_lccdf(time[i] | Loc[i], eAnc[i]);

}

} else if(dist == 5) { // Log-logistic distribution

for(i in 1:N) {

if(event[i] == 1)

target += loglogistic_lpdf(time[i] | eLoc[i], eAnc[i]);

else

target += loglogistic_lccdf(time[i] | eLoc[i], eAnc[i]);

}

} else if(dist == 6) { // Generalized gamma distribution

for(i in 1:N) {

if(event[i] == 1)

target += gengamma_lpdf(time[i] | Loc[i], eAnc[i], Q);

else

target += gengamma_lccdf(time[i] | Loc[i], eAnc[i], Q);

}

}

}

generated quantities {

// split mus, ds, and deltas into those for loc and anc

vector[ns] mu1 = loc[1:ns];

vector[ns] mu2 = anc[1:ns];

vector[ntrt - 1] d1;

vector[ntrt - 1] d2;

for(k in 1:ntrt - 1){

d1[k] = d[2*k-1];

d2[k] = d[2*k];

}

vector[ns*(ntrt - 1)] delta1 = loc[(ns +1):(ns + ns*(ntrt - 1))];

vector[ns*(ntrt - 1)] delta2 = anc[(ns +1):(ns + ns*(ntrt - 1))];

// split sigma into its components

vector[3] sigmaVec;

sigmaVec[1] = sigma[1,1];

sigmaVec[2] = sigma[1,2];

sigmaVec[3] = sigma[2,2];

// log likelihood for use with loo package

vector[N] log_lik;

if(dist == 1) { // Weibull

for(i in 1:N) {

if(event[i] == 1)

log_lik[i] = weibull_lpdf(time[i] | eAnc[i],

pow(eLoc[i], -1/eAnc[i]));

else

log_lik[i] = weibull_lccdf(time[i] | eAnc[i],

pow(eLoc[i], -1/eAnc[i]));

}

} else if(dist == 2) { // Gompertz (no stan function for this)

for(i in 1:N) {

if(event[i] == 1)

log_lik[i] = gompertz_lpdf(time[i] | eLoc[i], Anc[i]);

else

log_lik[i] = gompertz_lccdf(time[i] | eLoc[i], Anc[i]);

}

} else if(dist == 3) { // Gamma distribution

for(i in 1:N) {

if(event[i] == 1)

log_lik[i] = gamma_lpdf(time[i] | eAnc[i], eLoc[i]);

else

log_lik[i] = gamma_lccdf(time[i] | eAnc[i], eLoc[i]);

}

} else if(dist == 4) { // Log-normal distribution

for(i in 1:N) {

if(event[i] == 1)

log_lik[i] = lognormal_lpdf(time[i] | Loc[i], eAnc[i]);

else

log_lik[i] = lognormal_lccdf(time[i] | Loc[i], eAnc[i]);

}

} else if(dist == 5) { // Log-logistic distribution

for(i in 1:N) {

if(event[i] == 1)

log_lik[i] = loglogistic_lpdf(time[i] | eLoc[i], eAnc[i]);

else

log_lik[i] = loglogistic_lccdf(time[i] | eLoc[i], eAnc[i]);

}

} else if(dist == 6) { // Generalized gamma distribution

for(i in 1:N) {

if(event[i] == 1)

log_lik[i] = gengamma_lpdf(time[i] | Loc[i], eAnc[i], Q);

else

log_lik[i] = gengamma_lccdf(time[i] | Loc[i], eAnc[i], Q);

}

}

}

##### Figure A1: Kaplan-Meier survival curves for simulated event-times, for each treatment (colors) in each study (panels). Censored events are marked with a cross (“+”).


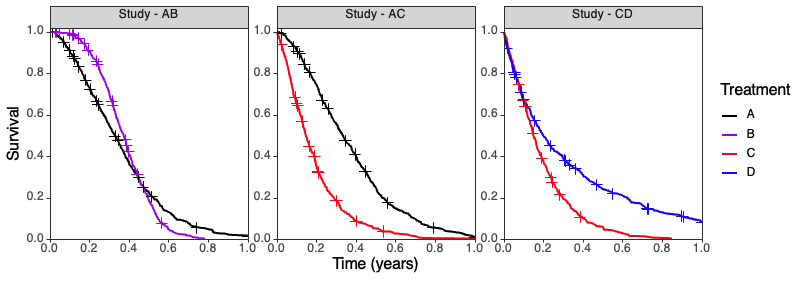


##### Figure A2: Top panels plot estimated survivals (with study AB as reference) over time for the simulated data set based on Weibull, Gompertz, gamma, log-normal and log-logistic FE models. Lower panels plot the estimated hazard ratios (relative to treatment A).


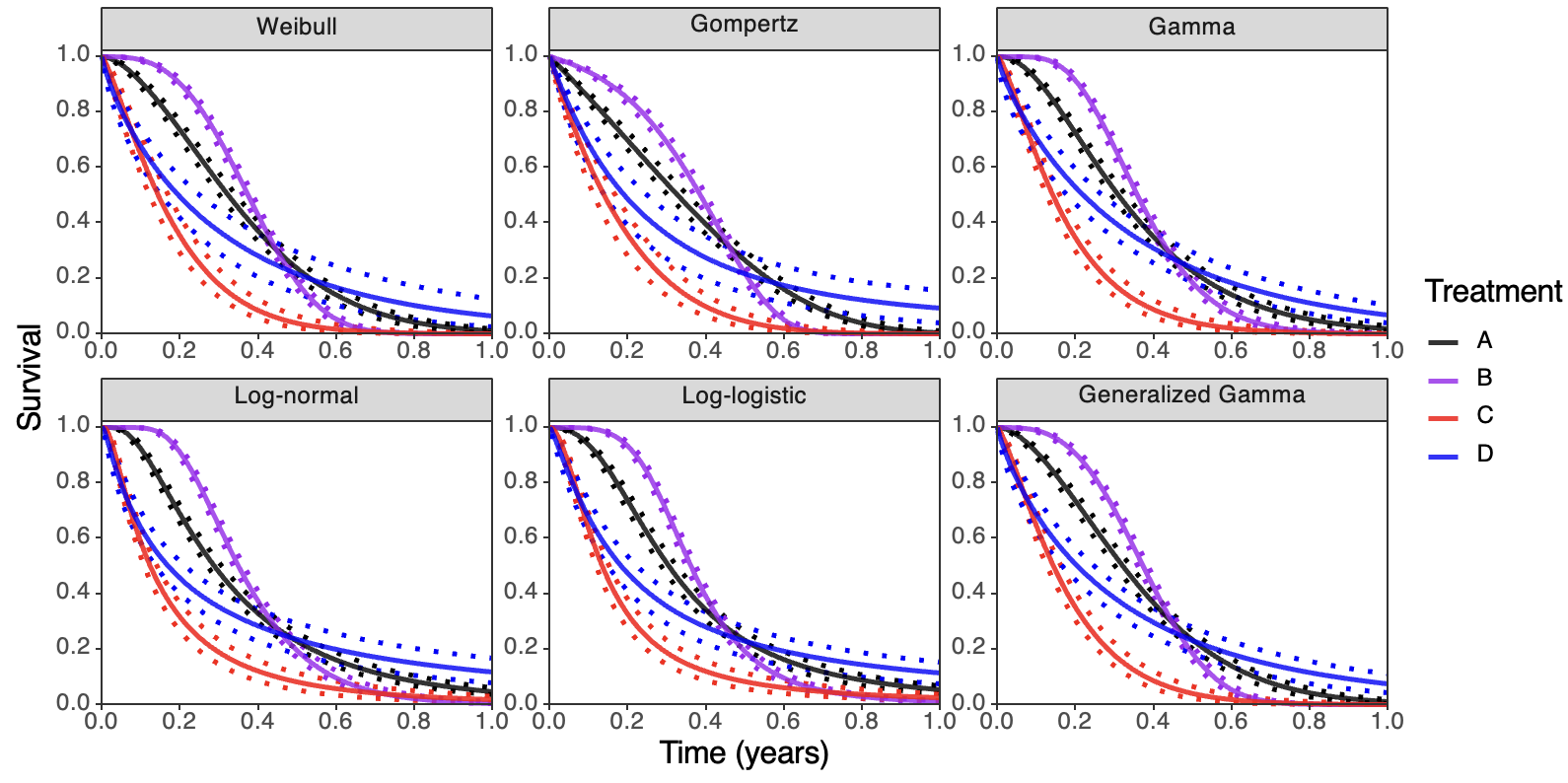


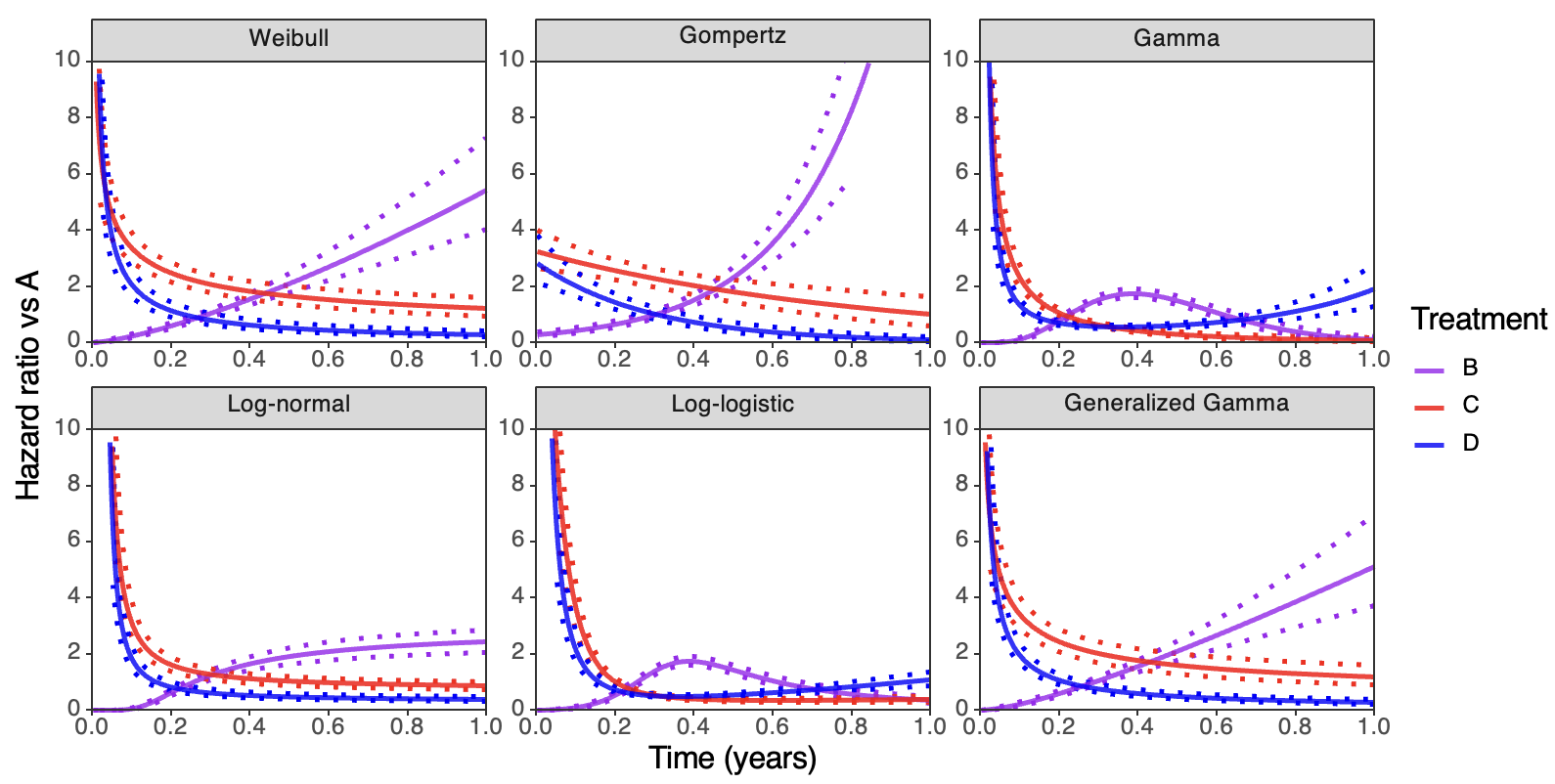


##### Table A1. Comparison of network meta-analysis parameter estimates versus study-specific estimates obtained from flexsurv

|  |  | **Weibull** | | **Gompertz** | | **gamma** | | **log-normal** | | **log-logistic** | | **gen. gamma** | |
| --- | --- | --- | --- | --- | --- | --- | --- | --- | --- | --- | --- | --- | --- |
|  |  | **NMA** | **Flexsurv estimate** | **NMA** | **Flexsurv estimate** | **NMA** | **Flexsurv estimate** | **NMA** | **Flexsurv estimate** | **NMA** | **Flexsurv estimate** | **NMA** | **Flexsurv estimate** |
| Study AB | $\mu_{1,1}$ | 1.544  (1.422, 1.665) | 1.543  (1.416, 1.662) | 0.351  (0.194, 0.509) | 0.355  (0.197, 0.514) | 1.889  (1.754, 2.020) | 1.893  (1.759, 2.027) | -1.246  (-1.312, -1.180) | -1.246  (-1.311, -1.180) | -1.188  (-1.252, -1.123) | -1.188  (-1.251, -1.125) | -0.932  (-0.994, -0.871) | -0.945  (-1.020, -0.870) |
|  | $\mu_{2,1}$ | 0.524  (0.454, 0.595) | 0.524  (0.451, 0.593) | 2.284  (1.911, 2.646) | 2.279  (1.897, 2.661) | 0.865  (0.742, 0.983) | 0.868  (0.749, 0.987) | -0.295  (-0.359, -0.229) | -0.297  (-0.360, -0.233) | 0.885  (0.810, 0.957) | 0.887  (0.813, 0.962) | -0.509  (-0.581, -0.436) | -0.501  (-0.580, -0.423) |
|  | $d_{1,2}$ | 1.102  (0.885, 1.318) | 1.101  (0.886, 1.317) | -1.278  (-1.545, -1.008) | -1.276  (-1.547, -1.005) | 1.003  (0.823, 1.189) | 1.004  (0.818, 1.189) | 0.196  (0.121, 0.270) | 0.196  (0.120, 0.271) | 0.171  (0.098, 0.242) | 0.172  (0.100, 0.244) | 0.058  (-0.006, 0.121) | 0.064  (-0.004, 0.131) |
|  | $d_{2,2}$ | 0.595  (0.496, 0.693) | 0.595  (0.495, 0.692) | 4.247  (3.591, 4.852) | 4.240  (3.581, 4.899) | 1.051  (0.885, 1.222) | 1.053  (0.882, 1.224) | -0.580  (-0.668, -0.489) | -0.581  (-0.671, -0.491) | 0.605  (0.500, 0.707) | 0.603  (0.497, 0.709) | -0.596  (-0.692, -0.494) | -0.596  (-0.694, -0.498) |
| Study AC | $\mu_{1,2}$ | 1.467  (1.349, 1.584) | 1.464  (1.344, 1.579) | 0.318  (0.160, 0.469) | 0.317  (0.157, 0.476) | 1.817  (1.684, 1.943) | 1.817  (1.684, 1.950) | -1.226  (-1.295, -1.157) | -1.226  (-1.294, -1.158) | -1.164  (-1.230, -1.098) | -1.164  (-1.229, -1.099) | -0.903  (-0.968, -0.839) | -0.908  (-0.983, -0.833) |
|  | $\mu_{2,2}$ | 0.507  (0.437, 0.577) | 0.505  (0.433, 0.574) | 2.210  (1.845, 2.574) | 2.217  (1.847, 2.587) | 0.823  (0.703, 0.934) | 0.823  (0.706, 0.941) | -0.259  (-0.324, -0.195) | -0.262  (-0.325, -0.198) | 0.861  (0.785, 0.934) | 0.862  (0.787, 0.936) | -0.490  (-0.563, -0.414) | -0.488  (-0.568, -0.409) |
|  | $d_{1,3}$ | 0.500  (0.318, 0.683) | 0.503  (0.326, 0.689) | 1.178  (0.977, 1.387) | 1.178  (0.972, 1.384) | 0.184  (0.001, 0.385) | 0.191  (0.000, 0.383) | -0.844  (-0.960, -0.733) | -0.844  (-0.958, -0.730) | -0.818  (-0.926, -0.711) | -0.820  (-0.926, -0.713) | -0.734  (-0.822, -0.634) | -0.734  (-0.831, -0.637) |
|  | $d_{2,3}$ | -0.306  (-0.404, -0.208) | -0.305  (-0.403, -0.207) | -1.170  (-1.841, -0.543) | -1.172  (-1.818, -0.526) | -0.494  (-0.654, -0.324) | -0.489  (-0.653, -0.326) | 0.302  (0.212, 0.391) | 0.302  (0.213, 0.391) | -0.280  (-0.383, -0.174) | -0.278  (-0.384, -0.173) | 0.303  (0.208, 0.398) | 0.303  (0.205, 0.401) |
| Study CD | $\mu_{1,3}+d_{1,3}$ | 1.941  (1.802, 2.079) | 1.939  (1.796, 2.080) | 1.422  (1.282, 1.555) | 1.424  (1.288, 1.559) | 1.950  (1.812, 2.086) | 1.953  (1.817, 2.090) | -2.076  (-2.171, -1.981) | -2.077  (-2.172, -1.982) | -1.973  (-2.064, -1.888) | -1.975  (-2.063, -1.886) | -1.627  (-1.714, -1.540) | -1.596  (-1.704, -1.489) |
|  | $\mu_{2,3}+d_{2,3}$ | 0.193  (0.124, 0.262) | 0.193  (0.121, 0.262) | 1.384  (0.817, 1.958) | 1.384  (0.832, 1.936) | 0.287  (0.175, 0.400) | 0.291  (0.179, 0.404) | 0.082  (0.021, 0.145) | 0.081  (0.018, 0.143) | 0.540  (0.466, 0.610) | 0.540  (0.466, 0.615) | -0.177  (-0.248, -0.103) | -0.195  (-0.276, -0.114) |
|  | $d_{1,4}{- d}_{1,3}$ | -1.032  (-1.207, -0.857) | -1.031  (-1.215, -0.857) | -0.127  (-0.321, 0.071) | -0.127  (-0.321, 0.067) | -1.214  (-1.432, -1.006) | -1.213  (-1.426, -0.999) | 0.318  (0.147, 0.494) | 0.320  (0.147, 0.492) | 0.308  (0.148, 0.470) | 0.306  (0.146, 0.466) | 0.486  (0.348, 0.624) | 0.496  (0.355, 0.637) |
|  | $d_{2,4}{- d}_{2,3}$ | -0.389  (-0.492, -0.286) | -0.390  (-0.494, -0.291) | -2.286  (-2.970, -1.581) | -2.286  (-2.971, -1.602) | -0.565  (-0.730, -0.416) | -0.567  (-0.726, -0.409) | 0.401  (0.308, 0.492) | 0.400  (0.308, 0.492) | -0.412  (-0.519, -0.307) | -0.411  (-0.519, -0.304) | 0.393  (0.291, 0.492) | 0.388  (0.285, 0.492) |
|  | $Q$ |  |  |  |  |  |  |  |  |  |  | 0.938  (0.841, 1.036) | Study AB:  0.896  (0.734, 1.058) |
|  |  |  |  |  |  |  |  |  |  |  |  |  | Study AC:  0.921  (0.763, 1.080) |
|  |  |  |  |  |  |  |  |  |  |  |  |  | Study CD:  1.008  (0.827, 1.189) |
